# Circulating Total Osteocalcin Reflects Bone Mineral Physiology Rather than Metabolic Risk in Pediatric Obesity

**DOI:** 10.3390/nu18091324

**Published:** 2026-04-22

**Authors:** Jakub Krzysztof Nowicki, Michał Kalisiak, Elżbieta Woźniak, Elżbieta Jakubowska-Pietkiewicz

**Affiliations:** 1 Department of Pediatrics, Neonatal Pathology and Metabolic Bone Diseases, Medical University of Lodz, 91-738 Lodz, Poland; elzbieta.wozniak@umed.lodz.pl (E.W.); elzbieta.jakubowska-pietkiewicz@umed.lodz.pl (E.J.-P.); 2 Faculty of Medicine, Medical University of Lodz, 91-738 Lodz, Poland; michal.kalisiak@stud.umed.lodz.pl

**Keywords:** osteocalcin, pediatric obesity, bone metabolism, body composition, children and adolescents

## Abstract

**Background:** Osteocalcin is a bone-derived protein traditionally regarded as a marker of bone formation, but experimental and clinical studies have suggested potential endocrine effects on energy and glucose metabolism. In pediatric populations, particularly in the context of obesity, the relationships between circulating osteocalcin, adiposity, and metabolic health remain inconsistent and poorly defined. **Objective:** To investigate associations between serum total osteocalcin and anthropometric, metabolic, biochemical, and body composition parameters in children and adolescents with obesity, with particular emphasis on adiposity and mineral metabolism. **Methods:** This retrospective cross-sectional study included 155 children and adolescents aged 4–18 years with obesity. Anthropometric measurements, laboratory parameters, and body composition assessed by dual-energy X-ray absorptiometry were extracted from medical records. Associations between osteocalcin z-scores and clinical variables were evaluated using linear regression models. Multivariable and extended regression models were applied to assess independent associations. **Results:** Osteocalcin was positively associated with markers of mineral metabolism, including serum 25-hydroxyvitamin D_3_ (β = 0.19, *p* = 0.012), serum calcium (β = 0.19, *p* = 0.015), and free triiodothyronine (β = 0.32, *p* < 0.001) in multivariable analyses. No independent associations were observed between osteocalcin and measures of adiposity, including body mass index, visceral adipose tissue index, leptin, or markers of glucose and lipid metabolism. **Conclusions:** In children and adolescents with obesity, circulating osteocalcin is primarily associated with mineral metabolism rather than adiposity or metabolic health. These findings support the interpretation of total osteocalcin as a clinically accessible marker of bone turnover and mineral homeostasis rather than a robust surrogate of metabolic dysfunction in pediatric obesity.

## 1. Introduction

Childhood obesity represents a major global public health challenge, with increasing prevalence worldwide and substantial short- and long-term consequences for metabolic, cardiovascular, and endocrine health [1,2]. In pediatric populations, excess adiposity is associated with early disturbances in glucose metabolism, dyslipidemia, low-grade inflammation, and alterations in hormonal regulation, potentially tracking into adulthood [3]. Despite extensive research, the complex interactions between adipose tissue, bone metabolism, and endocrine function during growth remain incompletely understood.

Osteocalcin (OC), a non-collagenous protein synthesized by osteoblasts, has traditionally been regarded as a marker of bone formation. In recent years, however, OC has attracted considerable attention due to its proposed endocrine functions extending beyond the skeleton [4]. Experimental studies, primarily in animal models, have suggested that osteocalcin may influence glucose metabolism, insulin sensitivity, and energy homeostasis, raising the hypothesis that bone may act as an endocrine organ involved in metabolic regulation [5,6]. In humans, observational studies have reported associations between circulating osteocalcin concentrations and markers of adiposity, insulin resistance, and lipid metabolism, although results have been inconsistent [7,8,9,10,11].

Several animal models have failed to demonstrate a consistent relationship between osteocalcin and the regulation of glucose and lipid metabolism, highlighting substantial heterogeneity of findings depending on the experimental model, genetic background, age, and metabolic status of the animals [12]. Likewise, results from population-based studies in humans are inconsistent—some observational analyses suggest report null findings or attenuation of these associations after adjustment for confounding factors [13,14,15]. Collectively, these discrepancies underscore the complexity of the bone—metabolism axis and the need for cautious interpretation of existing data.

Osteocalcin circulates in the blood in several molecular forms, including total, carboxylated, and undercarboxylated osteocalcin, each of which may reflect distinct biological processes. While carboxylated osteocalcin is mainly related to bone mineralization and undercarboxylated osteocalcin has been suggested to exert endocrine effects on glucose and energy metabolism, many clinical and epidemiological studies rely on measurements of total osteocalcin [16].

Therefore, the aim of the present study was to investigate the associations between serum total OC and a broad range of anthropometric, metabolic, biochemical, and body composition parameters in children and adolescents with obesity. In particular, we sought to assess whether OC is independently associated with markers of adiposity, including visceral adipose tissue index, as well as with parameters of mineral metabolism and renal function.

## 2. Materials and Methods

We conducted a retrospective cross-sectional review of medical records of children and adolescents hospitalized in the Department of Paediatrics, Neonatal Pathology, and Metabolic Bone Diseases at the University Pediatric Center, Central Clinical Hospital of the Medical University of Lodz, with diagnosed obesity between 1 January 2023 and 30 June 2025.

All procedures performed in this study involving human participants were in accordance with the ethical standards of the Ethics Committee of the Medical University of Lodz and the 1964 Helsinki declaration and its later amendments or comparable ethical standards. Ethical approval for all experimental protocols was applied for and granted by the Ethics Committee of the Medical University of Lodz: RNN/138/25/KE on 15 April 2025. The approval covered the analysis of collected routine clinical data extracted from medical records, without any additional study-specific interventions or procedures.

All patients and their legal guardians gave their written informed consent to participate in the study.

### 2.1. Study Design and Participants

**Figure 1:**
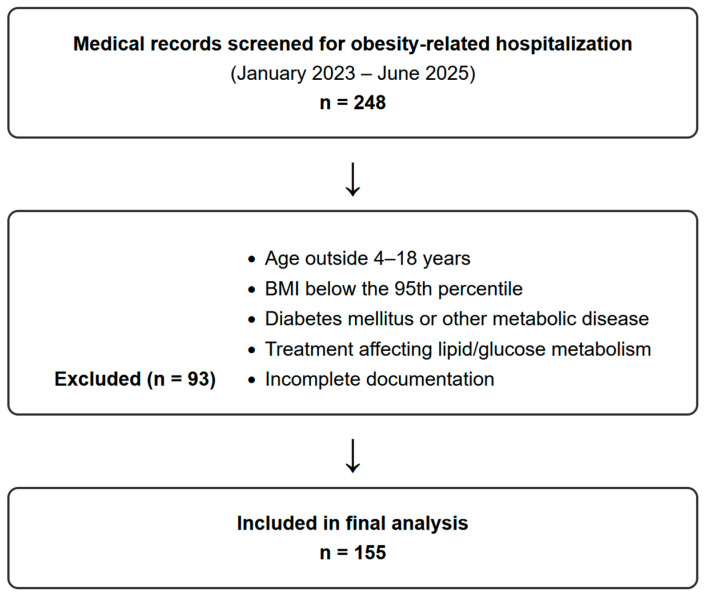
Flow chart of participant screening and selection. Medical records of 248 patients hospitalized for obesity-related evaluation between January 2023 and June 2025 were screened. After applying exclusion criteria, 155 children and adolescents were included in the final analysis.

The inclusion criteria for the study were as follows: age between 4 and 18 years and body mass index (BMI) above the 95th percentile according to age- and sex-specific percentile charts for the Polish population (Figure 1).

The exclusion criteria were as follows: comorbidities significantly affecting development or other metabolic diseases, including diabetes mellitus, as well as the use of medications known to affect lipid metabolism, such as metformin or glucagon-like peptide-1 (GLP-1) receptor antagonists, and incomplete documentation (Figure 1).

### 2.2. Anthropometric and Body Composition Assessment

Based on the above criteria, a group of 155 patients was selected (Figure 1). From the medical records, the following data were obtained: demographic (date of birth, sex); anthropometry (body weight, height); laboratory assessment (serum concentrations of OC, creatinine, calcium, 25-hydroxyvitamin D (25(OH)D_3_), parathyroid hormone (PTH), free triiodothyronine (fT3), ALT (alanine aminotransferase), leptin, lipid profile, glucose, glycated hemoglobin (HbA1c), insulin, eGFR, urea); and the dual-energy X-ray absorptiometry (DXA) results (visceral adipose tissue (VAT), lean mass index z-score, total body less head (TLBH) bone mineral density (BMD) z-score).

Height was measured using a stadiometer in a standing position, accurate to 1 cm. Body weight was measured using a SECA 756 (seca, Hamburg, Germany) with an accuracy of 100 g. The body mass index (BMI) was calculated using the standard formula: BMI = body weight [kg]/(height [m])^2^. These measurements were plotted on age- and sex-appropriate percentile charts developed for the Polish population in the OLA and OLAF projects were applied [17,18,19]. The percentiles were then converted to z-scores.

The DXA was performed using a Hologic Horizon WI (Hologic, Inc., Marlborough, MA, USA) densitometer. The visceral adipose tissue index (VATI) was calculated by dividing VAT mass obtained from DXA by height squared (m^2^).

### 2.3. Laboratory Measurements

OC concentration was tested using an electrochemiluminescence immunoassay (Roche, Basel, Switzerland), and 25(OH)D_3_ concentration was tested using a chemiluminescent microparticle immunoassay (Abbott, Dublin, Ireland). The remaining tests were performed using standard methods at the laboratory of the University Pediatric Center Medical University of Łódź.

OC and leptin levels were standardized, taking into account the reference ranges appropriate for the sex and age of the patients. The upper and lower reference values were set at +2 and −2 standard deviations, respectively. The reference ranges for the studied parameters were consistent with the guidelines of the Central Clinical Hospital of the Medical University of Łódź, where the tests were performed. Using the Schwartz formula, the glomerular filtration rate (GFR) was calculated for each patient based on serum creatinine levels and the patient’s height measurement [20].

### 2.4. Statistical Analysis

Statistical analyses were performed using Statistica software, version 13 (StatSoft, Tulsa, OK, USA). Continuous variables were assessed for normality using the Shapiro–Wilk test and visual inspection of distribution plots. Normally distributed data are presented as mean ± standard deviation, whereas non-normally distributed data are expressed as median and interquartile range. Categorical variables are presented as number and percentage.

Participants were stratified into tertiles according to serum OC concentration. The tertile-based analysis was intended as a descriptive comparison of crude osteocalcin distribution, whereas all inferential regression models were subsequently based on age- and sex-standardized osteocalcin z-scores treated as a continuous variable.

Tertiles were used to ensure adequate sample size within each subgroup. Comparisons across OC tertiles were performed using one-way analysis of variance (ANOVA) for normally distributed continuous variables, the Kruskal–Wallis test for non-normally distributed variables, and the χ^2^ test for categorical variables.

When a statistically significant overall difference was observed, post hoc pairwise comparisons were conducted using Bonferroni correction. All statistical tests were two-sided, and a *p* value < 0.05 was considered statistically significant. Exact *p* values are reported where appropriate.

Given the strong age- and sex-dependence of circulating OC concentrations during growth, OC values were standardized using age- and sex-specific reference data and expressed as z-scores. OC z-scores were used in subsequent analyses to assess associations with anthropometric, metabolic, biochemical, and body composition parameters independent of age and sex.

Associations between OC z-score and continuous outcome variables were evaluated using linear regression models, with OC z-score entered as a continuous independent variable. Regression results are presented as β coefficients with 95% confidence intervals, representing the change in the outcome variable per one standard deviation increase in OC z-score. All statistical tests were two-sided, and a *p* value < 0.05 was considered statistically significant.

To further evaluate the independent associations between OC and biochemical parameters, multivariable linear regression analyses were performed. The OC z-score was included as the independent variable. Outcome variables entered into the multivariable models were selected a priori based on statistical significance in univariable analyses.

To assess whether markers of adiposity and metabolic health provided additional explanatory value beyond the primary mineral metabolism model, we constructed a series of extended multivariable regression models. Each model was based on the core mineral metabolism variables (25-hydroxyvitamin D_3_, serum calcium, and fT3), with one additional metabolic or body composition parameter added separately. This approach was intended to test whether adiposity-related markers materially improved model fit or altered the observed osteocalcin associations.

Model performance was assessed using the adjusted coefficient of determination (adjusted R^2^), and overall model significance was evaluated using the corresponding model *p* value. Regression coefficients (β) with 95% confidence intervals and *p* values are reported for the added variable in each extended model.

## 3. Results

Participants were stratified into tertiles according to serum OC concentration (Table 1). Significant differences in age and sex distribution were observed across tertiles (*p* < 0.001). BMI z-score differed across tertiles, with post hoc analyses indicating higher BMI z-scores in the lowest OC tertile compared with the highest tertile (*p* = 0.02).

Marked differences were found in markers of bone and mineral metabolism. Serum 25(OH)D_3_ concentrations differed significantly across tertiles (*p* = 0.03) and increased progressively with higher OC levels. Vitamin D_3_ deficiency was identified in 62 patients (40%). Serum calcium concentrations were lower in the lowest tertile compared with the middle and highest tertiles, while no significant differences were observed between the latter two groups.

fT3 concentrations differed across tertiles (*p* < 0.001), with post hoc analyses demonstrating higher fT3 levels in the highest and middle OC tertiles compared with the lowest tertile (*p* < 0.001). Participants in the lowest OC tertile exhibited significantly higher leptin z-scores compared with those in the middle and highest tertiles (*p* < 0.001).

Similarly, VATI differed significantly across tertiles (*p* < 0.001), with post hoc analyses revealing greater visceral adiposity in the lowest tertile compared with the other groups, whereas no significant differences were observed between the middle and highest tertiles. TLBH BMD z-score and LMI z-score did not differ significantly across OC tertiles.

eGFR was significantly lower in the lowest OC tertile compared with the middle and highest tertiles (*p* < 0.001), with no significant difference between the latter two groups. No significant differences were observed across OC tertiles in lipid profile parameters, fasting plasma glucose, glycated hemoglobin, fasting insulin, urea concentrations, or alanine aminotransferase activity.

Linear regression analyses using OC z-score as a continuous predictor are presented in Table 2. Higher OC z-scores were significantly positively associated with 25(OH)D_3_ (β = 0.26, 95% CI 0.11 to 0.42; *p* = 0.001), fT3 (β = 0.41, 95% CI 0.26 to 0.56; *p* < 0.001), serum calcium concentrations (β = 0.29, 95% CI 0.14 to 0.45; *p* < 0.001), and eGFR (β = 0.18, 95% CI 0.02 to 0.34; *p* < 0.03).

In contrast, no significant associations were observed between OC z-score and anthropometric measures of adiposity, including BMI z-score, leptin z-score, or VATI.

Results of the multivariable linear regression analyses are presented in Table 3. After simultaneous inclusion of outcomes that were significant in univariable analyses, OC z-score remained independently associated with several biochemical parameters.

**Figure 2:**
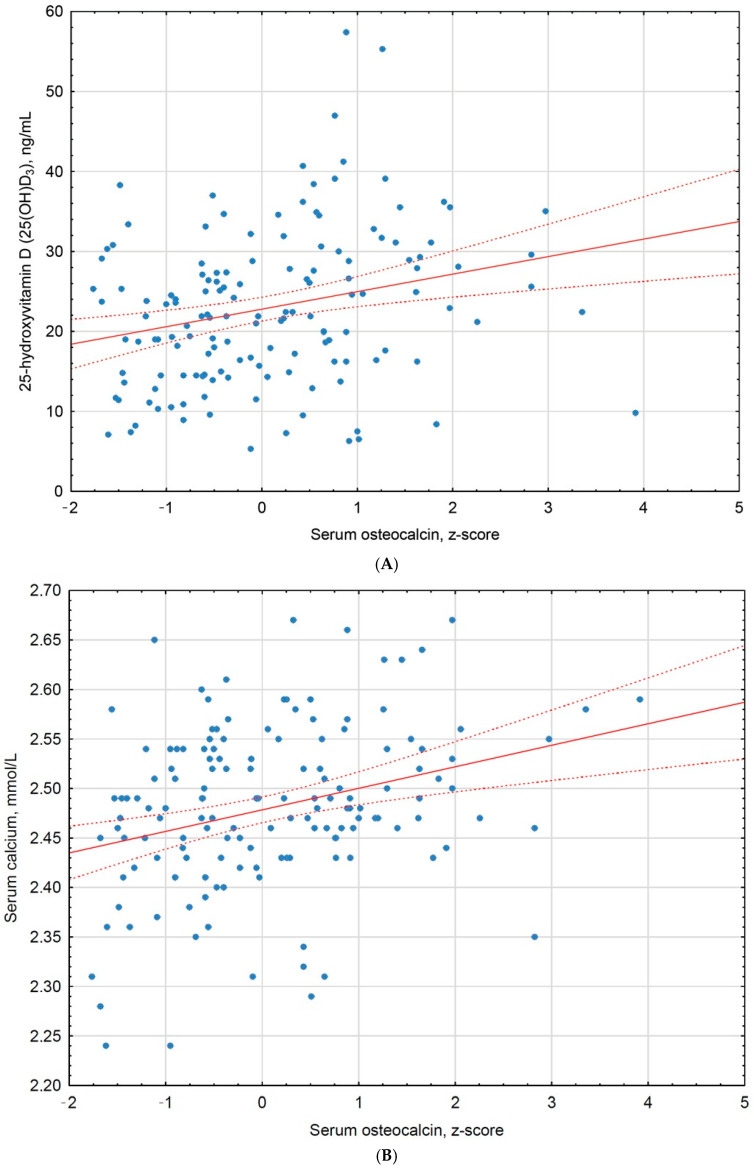

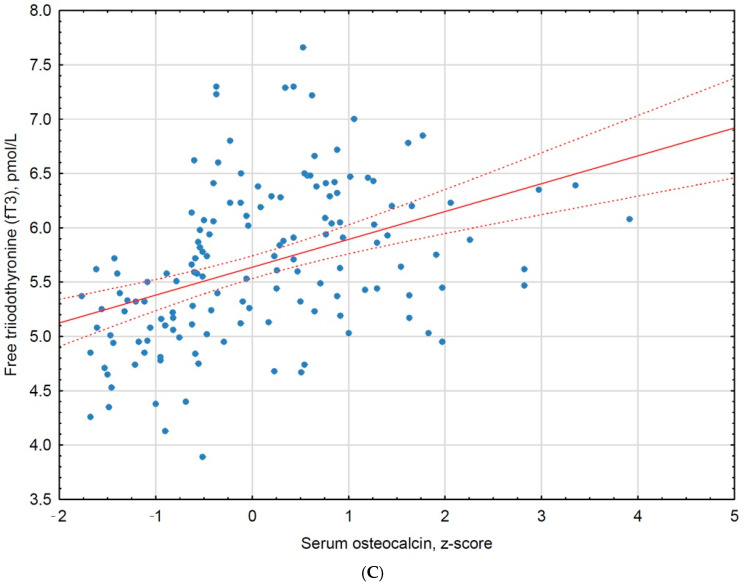
Scatter plots illustrating associations between osteocalcin z-score and (**A**) serum 25-hydroxyvitamin D, (**B**) serum calcium, and (**C**) free triiodothyronine. Solid lines indicate linear regression fits and dashed lines denote 95% confidence intervals. Osteocalcin concentrations were standardized to age- and sex-specific z-scores.

Higher OC z-scores were positively associated with 25(OH)D_3_ (β = 0.19; 95% CI: 0.04 to 0.33; *p* = 0.012) (Figure 2A), serum calcium levels (β = 0.19; 95% CI: 0.04 to 0.34; *p* = 0.015) (Figure 2B), and fT3 concentrations (β = 0.32; 95% CI: 0.16 to 0.48; *p* < 0.001) (Figure 2C). The association between OC z-score and eGFR did not reach statistical significance in multivariable analysis (*p* = 0.91).

Exploratory sex-stratified analyses are provided in Appendix A (Table A1, Table A2, Table A3 and Table A4). The overall association pattern remained more pronounced in girls, whereas in boys, similar directional trends were observed without reaching statistical significance in multivariable models.

Results of the extended multivariable regression analyses are presented in Table 4.

All extended models were statistically significant (model *p* values < 0.001); however, adjusted R^2^ values were lower than those observed for the primary multivariable model presented in Table 3, indicating reduced overall model fit after inclusion of additional metabolic and body composition parameters. Moreover, none of the added variables—including leptin z-score, BMI z-score, glucose, insulin, VATI, or LMI—were independently associated with OC z-score after adjustment for 25(OH)D_3_, calcium and fT3 levels (all *p* values > 0.5).

## 4. Discussion

In this study, we evaluated the associations between circulating osteocalcin, expressed as age- and sex-standardized z-scores, and a broad range of anthropometric, metabolic, biochemical, and body composition parameters in children and adolescents with obesity. The principal finding is that osteocalcin z-score was consistently associated with markers of mineral metabolism, including serum 25-hydroxyvitamin D_3_ and serum calcium. In contrast, osteocalcin z-score was not independently associated with indices of adiposity, visceral fat accumulation, or markers of glucose and lipid metabolism.

The positive association between osteocalcin z-score and serum 25-hydroxyvitamin D_3_ observed in both univariable and multivariable analyses supports the concept of coordinated regulation within the bone mineral axis. The association between osteocalcin and 25-hydroxyvitamin D_3_ is also biologically plausible at the cellular level. Active vitamin D_3_ signaling promotes osteoblast differentiation and stimulates osteocalcin gene transcription via vitamin D_3_ receptor–dependent mechanisms, thereby increasing osteocalcin synthesis during active bone formation [21,22]. Similarly, the independent association between osteocalcin z-score and serum calcium further underscores the close link between circulating osteocalcin and mineral metabolism in pediatric populations. Together, these findings suggest that, in children and adolescents with obesity, circulating osteocalcin primarily reflects bone-related metabolic activity. Of note, population-based pediatric data also indicate that associations between 25(OH)D_3_ and bone turnover markers including osteocalcin may vary by calcium status and seasonality, underscoring the context-dependent nature of the vitamin D–bone marker relationship [23,24]. Interestingly, in pediatric chronic kidney disease, uncarboxylated osteocalcin has been reported to correlate negatively with 25(OH)D_3_ levels, a pattern attributed to the complex mineral bone disorder characteristic of CKD, where disturbed vitamin D_3_ metabolism and carboxylation processes influence OC fractions differently [25]. Given that our study assessed total osteocalcin in youth with preserved renal function, divergent associations with 25(OH)D_3_ may reflect population-specific differences in metabolism and the relative contribution of bone turnover versus renal/mineral dysfunction to circulating osteocalcin levels.

Free triiodothyronine (fT3) was independently and positively associated with osteocalcin z-score in our multivariable models. This finding is biologically plausible, as fT3 represents the metabolically active thyroid hormone and exerts direct effects on osteoblast differentiation, bone formation, and skeletal remodeling during growth [26,27,28].

We also observed a positive association between osteocalcin z-score and eGFR in univariable analyses; however, this association did not remain statistically significant after multivariable adjustment. This attenuation suggests that the relationship between osteocalcin and renal function may be partially mediated or confounded by mineral metabolism parameters. Recent clinical studies in patients with chronic kidney disease have shown that circulating osteocalcin concentrations are related to renal function markers, such as estimated GFR and serum creatinine. In a cohort of CKD adult patients, serum osteocalcin was associated with poorer renal function and bone metabolic disturbances [29], and elevated OC levels were negatively correlated with eGFR, consistent with reduced clearance and altered mineral handling in impaired renal function [30]. Consistent with the notion that renal function influences circulating osteocalcin concentrations, a recent systematic review and meta-analysis in diabetic kidney disease reported inverse associations between serum osteocalcin levels and kidney disease progression, with lower osteocalcin concentrations observed in more advanced stages of renal impairment, further supporting a link between osteocalcin dynamics and markers of renal function [31]. On the other hand, in pediatric cohorts with mild-to-moderate, relatively stable renal dysfunction, osteocalcin may show an inverse association with estimated GFR in simple correlation analyses; however, this relationship can disappear when within-subject variability and repeated measurements are accounted for, suggesting that osteocalcin levels are independent of filtration parameters [32]. Importantly, the aforementioned studies were conducted in populations with chronic kidney disease or other forms of renal impairment, in whom osteocalcin concentrations may be substantially influenced by reduced renal clearance and altered bone mineral metabolism. In contrast, the participants in the present study had preserved renal function, which may partly explain the weaker and non-independent associations between osteocalcin and eGFR observed after multivariable adjustment.

The lack of an independent association between osteocalcin z-score and total body less head bone mineral density z-score is consistent with the physiological distinction between bone turnover and bone mass. In a cohort of healthy children and adolescents, Gajewska et al. demonstrated positive correlations between osteocalcin and total body less head BMD and BMD Z-score exclusively in prepubertal children aged 5–10 years, while such relationships were not observed beyond this developmental stage [33]. In a cohort of healthy Finnish children and adolescents, circulating and urinary osteocalcin values were found to reflect growth status and bone turnover more accurately than bone mineral density or body composition [24]. Interestingly, in a cohort of Egyptian children aged 6 to 10 years, obese participants exhibited that osteocalcin was negatively correlated with BMD, suggesting that in the context of pediatric obesity, higher bone mineral accrual may be mechanically driven, while circulating osteocalcin reflects dynamic bone turnover processes that are dissociated from static densitometric measures [34].

Biochemical markers such as osteocalcin reflect dynamic processes of bone formation and resorption that vary markedly with growth and remodeling during childhood and adolescence, whereas DXA-derived BMD measures static bone mineral content at a given time point. Prior pediatric studies have demonstrated that serum osteocalcin and other bone turnover markers exhibit age- and puberty-dependent variability that does not necessarily parallel changes in BMD z-scores, underscoring that osteocalcin primarily reflects bone metabolic activity rather than static bone mineral accrual.

In contrast to some previous reports [35,36,37], we did not identify independent associations between osteocalcin and measures of adiposity or metabolic health, including body mass index z, visceral adipose tissue index, leptin, fasting glucose, insulin, or lipid profile parameters.

A cross-sectional study of obese adolescents by Lenders and colleagues [35] reported inverse associations between total osteocalcin and measures of visceral adiposity and body mass index, which remained significant after adjustment for age, sex, and other factors. In contrast, our analysis included children and adolescents with obesity across a broad developmental spectrum, ranging from early childhood to late adolescence (4–17 years), and used age- and sex-standardized OC z-scores. Within this heterogeneous pediatric population, we did not observe independent associations between OC z-score and indices of adiposity or metabolic health in fully adjusted models, suggesting that developmental stage and methodological differences may contribute to the divergent findings across studies.

Several pediatric investigations have reported inverse associations between total osteocalcin and markers of metabolic risk, including leptin, insulin resistance indices, and lipid parameters, in children and adolescents with obesity. In cohorts analyzed by Garanty-Bogacka et al. and Jürimäe et al. [36,37], lower osteocalcin concentrations were associated with adverse metabolic profiles, while Wang et al. [38] demonstrated that such relationships were attenuated after adjustment for developmental and clinical covariates. Notably, these studies predominantly evaluated raw osteocalcin concentrations in narrower age ranges or specific pubertal subgroups. In contrast, our use of age- and sex-standardized osteocalcin z-scores across a broad pediatric age spectrum, combined with extended multivariable models, may have minimized residual confounding related to growth and maturation, thereby contributing to the absence of independent associations between osteocalcin and metabolic parameters in our cohort.

Notably, analysis of longitudinal data published by Roswall et al. [39] indicate that low cord blood osteocalcin and vitamin D concentrations at birth were associated with higher BMI and impaired glucose homeostasis in early childhood. This underscores the multifactorial etiology of childhood overweight and obesity, where socioeconomic, parental, early life, and contextual neighborhood factors influence weight trajectories from infancy to early childhood. In the study, lower area-level purchasing power, parental BMI, and rapid weight gain in infancy were associated with elevated adiposity in preschool-aged children, suggesting that early environmental and familial conditions play critical roles in shaping later obesity risk [39]. These observations highlight that metabolic and hormonal markers alone—such as osteocalcin—may capture only a small part of the complex biology underlying childhood obesity.

On the other hand, some pediatric and adolescent studies have similarly reported null or weak associations between osteocalcin and leptin/obesity-related parameters, supporting the interpretation that osteocalcin is not a universal surrogate marker of metabolic health in youth.

In a pediatric cohort analyzed by Guidici et al. [40], total osteocalcin showed only weak inverse correlations with leptin in unadjusted analyses, all of which disappeared after further adjustment. In that study, total osteocalcin was not independently related to glucose or other biochemical outcomes, while weak associations involving uncarboxylated osteocalcin emerged only after sequential adjustment and were highly model-dependent [40].

A study by Tubič et al. [41] in prepubertal children (aged 2–9 years) reported that none of the measured osteocalcin forms—total, carboxylated, or undercarboxylated—were significantly correlated with anthropometric measures or metabolic parameters, including insulin, glucose, and indices of insulin resistance, despite small group differences in carboxylated osteocalcin [41]. This lack of association in young children reinforces the notion that osteocalcin’s relationships with adiposity and energy metabolism may be weak or absent in early childhood.

Pubertal maturation provides an important physiological framework for interpreting circulating osteocalcin in pediatric obesity [42]. Bone turnover markers, including osteocalcin, follow the dynamic pattern of skeletal growth, remaining relatively stable during mid-childhood and rising again during the pubertal growth spurt, with sex-specific timing related to maturation tempo [43]. Importantly, the reference standards used in our study were adjusted for both age and sex, thereby inherently accounting for the natural differences in pubertal timing and maturation velocity between boys and girls.

Exploratory sex-stratified analyses suggested that the association pattern may be more pronounced in girls, in whom independent relationships with 25-hydroxyvitamin D and fT3 persisted in multivariable models, whereas in boys the direction of effects remained broadly similar but did not reach statistical significance, likely reflecting reduced subgroup power and greater heterogeneity of pubertal maturation. Notably, in girls we also observed univariable associations between osteocalcin z-score and both visceral adipose tissue index and lean mass index, although these relationships were no longer apparent after multivariable adjustment. Nevertheless, this remains an interesting observation in the context of recent longitudinal pediatric data by Berggren et al. [44], who reported sex-dependent associations between early-life osteocalcin concentrations and later body composition trajectories, particularly stronger links with fat mass development in girls.

Taken together, the available pediatric evidence suggests that associations between circulating osteocalcin and adiposity or metabolic health are heterogeneous, highly context-dependent, and strongly influenced by developmental stage, body composition, and analytical approach. While inverse relationships between osteocalcin and metabolic risk markers have been reported in selected cohorts—often in narrower age ranges or using raw osteocalcin concentrations—other studies, including those employing more extensive adjustment strategies or focusing on younger children, have demonstrated weak or null associations. In this context, our findings support the interpretation that, in children and adolescents with obesity, osteocalcin does not function as a robust or universal surrogate marker of metabolic health. Rather, variability in circulating osteocalcin appears to predominantly reflect bone—mineral physiology.

Extended multivariable models further supported this interpretation. The addition of metabolic and body composition parameters, including BMI z-score, leptin, fasting glucose, fasting insulin, visceral adipose tissue index, and lean mass index, did not materially improve model performance and did not attenuate the associations between osteocalcin and mineral metabolism markers.

An important strength of the present study is the comprehensive phenotyping of a relatively large pediatric obesity cohort, including DXA-derived visceral adiposity assessment and age- and sex- standardized osteocalcin z-scores. Within this well-characterized framework, our findings consistently indicate that circulating total osteocalcin is more closely linked to bone mineral physiology than to metabolic risk profiling in a population of obese children. These data contribute clinically relevant evidence suggesting that total osteocalcin may have limited utility as a surrogate biomarker of adiposity-related metabolic dysfunction in pediatric obesity.

However, certain limitations must also be considered. The retrospective and cross-sectional design precludes causal inference and limits the ability to assess temporal relationships between osteocalcin, mineral metabolism, and metabolic health. An important methodological consideration is that our analyses were based on total osteocalcin, which represents the fraction routinely available in standard clinical laboratory practice in our center. In contrast, undercarboxylated osteocalcin was not available in this retrospective cohort and may demonstrate different metabolic associations. However, this approach is in line with several previously cited pediatric and human observational studies, allowing direct comparison of our findings with the existing clinical literature. Pubertal stage was not directly assessed, and residual confounding related to pubertal development cannot be fully excluded despite age- and sex-standardization. Notably, the absence of associations between osteocalcin z-score and lean mass index or total body less head bone mineral density suggests that pubertal effects are unlikely to be the dominant drivers of the observed relationships. Furthermore, the findings may not be generalizable beyond children and adolescents with obesity or to populations with different ethnic or clinical characteristics.

## 5. Conclusions

In conclusion, in this relatively large cohort of children and adolescents with obesity, circulating total osteocalcin was more strongly associated with markers of bone mineral physiology than with adiposity-related metabolic risk. These findings support the interpretation of total osteocalcin as a clinically accessible marker of bone turnover and mineral homeostasis rather than a robust surrogate of metabolic dysfunction in pediatric obesity. However, the retrospective cross-sectional design precludes causal inference, and the exclusive assessment of total osteocalcin does not allow evaluation of potentially distinct roles of its molecular fractions. Further prospective studies are warranted to clarify the complex developmental interactions between bone metabolism, mineral homeostasis, and metabolic health across pediatric growth stages.

## Figures and Tables

**Table 1:** Clinical and biochemical characteristics of the study population according to crude osteocalcin tertiles.

Variable	Total (*n* = 155)	Tertile 1 (≤60 ng/mL), *n* = 53	Tertile 2 (61–97 ng/mL), *n* = 51	Tertile 3 (>97 ng/mL), *n* = 51	*p* Value
Age, years	13 (11; 15)	16 (14; 17)	11 (8; 14)	12 (10; 13)	<0.001
Male sex, n (%)	78 (50.3)	16 (30.2)	35 (68.6)	27 (52.9)	<0.001
Height z-score	1.04 (0.10; 1.88)	0.44 (−0.61; 1.55)	1.34 (0.50; 1.88)	1.13 (0.58; 2.05)	0.08
Body weight z-score	2.33 (1.88; 3.09)	3.09 (2.05; 3.09)	2.33 (1.88; 3.09)	2.33 (2.05; 3.09)	0.17
Body mass index (BMI) z-score	2.33 (2.05; 3.09)	3.09 (2.33; 3.09)	2.33 (1.88; 3.09)	2.33 (1.88; 3.09)	0.02
Osteocalcin, ng/mL	75 (42; 108)	35 (28; 42)	75 (68.5; 85)	124 (108; 147)	
25-hydroxyvitamin D (25(OH)D_3_), ng/mL	22.4 (16.2; 29.1)	19.0 (12.8; 24.2)	22.1 (17.9; 30.0)	26.6 (17.6; 35.0)	0.03
Serum calcium, mmol/L	2.48 (2.43; 2.54)	2.45 (2.40; 2.49)	2.50 (2.46; 2.55)	2.49 (2.46; 2.55)	0.02
Parathyroid hormone (PTH), pmol/L	5.62 (4.39; 7.28)	5.64 (4.49; 7.28)	5.16 (3.97; 6.90)	5.99 (5.05; 7.99)	0.09
Free triiodothyronine (fT3), pmol/L	5.66 ± 0.71	5.10 ± 0.45	5.95 ± 0.67	5.93 ± 0,64	<0.001
Alanine aminotransferase (ALT), U/L	21 (15; 27)	19 (15; 30)	22 (17; 27)	20 (15; 26)	0.36
Leptin z-score	2.8 (1.9; 3.6)	3.3 (2.4; 4.2)	2.3 (1.5; 3.6)	2.5 (1.9; 3.1)	<0.001
Total cholesterol, mmol/L	4.18 ± 0.75	4.12 ± 0.80	4.13 ± 0.67	4.29 ± 0.79	0.47
High-density lipoprotein cholesterol (HDL-C), mmol/L	1.14 (1.02; 1.29)	1.13 (0.99; 1.29)	1.21 (1.06; 1.34)	1.22 (0.99; 1.49)	0.30
Low-density lipoprotein cholesterol (LDL-C), mmol/L	2.99 ± 0.80	2.99 ± 0.89	2.86 ± 0.71	3.13 ± 0.78	0.21
Triglycerides, mmol/L	1.09 (0.74; 1.37)	1.07 (0.77; 1.35)	0.92 (0.68; 1.31)	1.10 (1.02; 1.29)	0.06
Fasting serum glucose, mmol/L	4.89 ± 0.38	4.88 ± 0.41	4.90 ± 0.45	4.91 ± 0.31	0.91
Glycated hemoglobin (HbA1c), %	5.3 (5.1; 5.5)	5.2 (5.0; 5.5)	5.4 (5.2; 5.6)	5.3 (5.3; 5.5)	0.18
Fasting insulin, pmol/L	74.6 (47.5; 101.3)	73.9 (50.2; 111.3)	68.7 (47.7; 81.6)	83.8 (44.4; 101.3)	0.10
eGFR (mL/min/1.73 m^2^)	115 ± 19.7	104 ± 16.1	124 ± 23.4	116 ± 13.0	<0.001
Urea (mmol/L)	0.35 (0.30; 0.40)	0.36 (0.32; 0.42)	0.32 (0.30; 0.39)	0.35 (0.31; 0.40)	0.15
Visceral adipose tissue index (VATI; g/m^2^)	224 (195; 278)	247 (218; 305)	222 (186; 269)	218 (184; 246)	<0.01
Lean mass index (LMI) z-score	0.92 (0.25; 1.55)	1.08 (0.67; 1.64)	0.55 (−0.10; 1.64)	0.82 (0.31; 1.41)	0.31
Total body less head (TLBH) bone mineral density (BMD) z-score	1.23 ± 1.16	1.08 ± 1.19	1.19 ± 1.20	1.42 ± 1.08	0.32

Z-scores were calculated based on age- and sex-specific reference values. Continuous variables with non-normal distribution are presented as median (interquartile range, Q1; Q3), normally distributed variables as mean ± standard deviation, and categorical variables as a number (percentage). Comparisons across OC tertiles were performed using one-way ANOVA for normally distributed variables, the Kruskal–Wallis test for non-normally distributed variables, and the χ^2^ test for categorical variables. A two-sided *p* value < 0.05 was considered statistically significant. OC tertiles were based on crude concentrations; age- and sex-standardized z-scores were used in all further analyses.

**Table 2:** Associations between osteocalcin z-score and clinical, anthropometric, metabolic, and body composition parameters.

Outcome Variable	β (95% CI)	*p* Value
Height z-score	0.13 (−0.03 to 0.29)	0.12
Body weight z-score	−0.04 (−0.20 to 0.12)	0.65
Body mass index (BMI) z-score	−0.10 (−0.26 to 0.06)	0.20
25-hydroxyvitamin D (25(OH)D_3_), ng/mL	0.26 (0.11 to 0.42)	0.001
Serum calcium, mmol/L	0.29 (0.14 to 0.45)	<0.001
Parathyroid hormone (PTH), pmol/L	0.13 (−0.03 to 0.29)	0.10
Free triiodothyronine (fT3), pmol/L	0.41 (0.26 to 0.56)	<0.001
Alanine aminotransferase (ALT), U/L	−0.04 (−0.20 to 0.12)	0.60
Leptin z-score	−0.10 (−0.26 to 0.07)	0.25
Total cholesterol, mmol/L	0.11 (−0.05 to 0.27)	0.16
High-density lipoprotein cholesterol (HDL-C), mmol/L	−0.008 (−0.17 to 0.15)	0.93
Low-density lipoprotein cholesterol (LDL-C), mmol/L	0.10 (−0.06 to 0.26)	0.22
Triglycerides, mmol/L	0.10 (−0.06 to 0.26)	0.24
Fasting serum glucose, mmol/L	−0.02 (−0.54 to 0.44)	0.84
Glycated hemoglobin (HbA1c), %	0.16 (−0.03 to 0.35)	0.10
Fasting insulin, pmol/L	−0.08 (−0.20 to 0.14)	0.72
eGFR (mL/min/1.73 m^2^)	0.18 (0.02 to 0.34)	0.03
Urea, mmol/L	0.08 (−0.08 to 0.24)	0.34
Visceral adipose tissue index (VATI; g/m^2^)	−0.11 (−0.27 to 0.05)	0.19
Lean mass index (LMI) z-score	−0.11 (−0.28 to 0.06)	0.20
Total body less head (TLBH) bone mineral density (BMD) z-score	0.10 (−0.06 to 0.26)	0.21

Linear regression analyses were performed with OC z-score as a continuous independent variable. β coefficients represent the change in the outcome variable per 1 SD increase in OC z-score. Z-scores were calculated using age- and sex-specific reference values.

**Table 3:** Multivariable linear regression analysis of associations between osteocalcin z-score and selected biochemical outcomes.

Outcome	β (95% CI)	*p* Value
25-hydroxyvitamin D (25(OH)D_3_), ng/mL	0.19 (0.04 to 0.33)	0.012
Serum calcium, mmol/L	0.19 (0.04 to 0.34)	0.015
Free triiodothyronine (fT3), pmol/L	0.32 (0.16 to 0.48)	<0.001
eGFR (mL/min/1.73 m^2^)	0.009 (−0.15 to 0.17)	0.91

Adjusted R^2^ = 0.21, *p* < 0.001 for model. Multivariable linear regression models were fitted with OC z-score as the independent variable. Outcome variables included parameters that were statistically significant in univariable analyses. β coefficients represent the change in the outcome variable per 1 standard deviation increase in OC z-score. *p* values < 0.05 were considered statistically significant.

**Table 4:** Multivariable linear regression models evaluating the association of osteocalcin z-score with additional covariates entered separately into the core multivariable model—metabolic and body composition parameters.

Model	Adjusted R^2^; *p* Value for Model	β (95% CI) for Added Variable	*p* Value for Added Variable
BMV + Body mass index (BMI) z-score	0.15; *p* < 0.001	0.02 (−0.13 to 0.18)	0.78
BMV + Leptin z-score	0.13; *p* < 0.001	−0.02 (−0.18 to 0.14)	0.81
BMV + Fasting serum glucose, mmol/L	0.14; *p* < 0.001	0.01 (−0.15 to 0.16)	0.94
BMV + Fasting insulin, pmol/L	0.15; *p* < 0.001	−0.03 (−0.19 to 0.13)	0.73
BMV + Visceral adipose tissue index (VATI) (g/m^2^)	0.15; *p* < 0.001	−0.05 (−0.20 to 0.10)	0.54
BMV + Lean mass index (LMI) z-score	0.13; *p* < 0.001	0.04 (−0.13 to 0.22)	0.62

Multivariable linear regression models were fitted with OC z-score as the independent variable. The base model included serum 25-hydroxyvitamin D_3_, serum calcium, and fT3 levels (BMV). Each row represents a sensitivity model built on the primary mineral metabolism framework (BMV), with one additional metabolic or body composition parameter entered separately to evaluate its incremental explanatory value.

## Data Availability

The original contributions presented in this study are included in the article. Further inquiries can be directed to the corresponding author.

## Appendix A

**Table A1:** Associations between osteocalcin z-score and clinical, anthropometric, metabolic, and body composition parameters in girls.

Outcome Variable	β (95% CI)	*p* Value
Height z-score	0.12 (−0.19 to 0.20)	0.95
Body weight z-score	−0.14 (−0.65 to 0.16)	0.23
Body mass index (BMI) z-score	−0.14 (−0.84 to 0.21)	0.24
25-hydroxyvitamin D (25(OH)D_3_), ng/mL	0.34 (0.01 to 0.66)	0.003
Serum calcium, mmol/L	0.31 (0.09 to 0.54)	0.006
Parathyroid hormone (PTH), pmol/L	0.12 (−0.13 to 0.34)	0.37
Free triiodothyronine (fT3), pmol/L	0.59 (0.40 to 0.77)	<0.001
Alanine aminotransferase (ALT), U/L	−0.02 (−0.24 to 0.23)	0.99
Leptin z-score	−0.10 (−0.34 to 0.13)	0.39
Total cholesterol, mmol/L	0.11 (−0.12 to 0.34)	0.37
High-density lipoprotein cholesterol (HDL-C), mmol/L	−0.1 (−0.33 to 0.14)	0.41
Low-density lipoprotein cholesterol (LDL-C), mmol/L	0.08 (−0.15 to 0.31)	0.49
Triglycerides, mmol/L	0.27 (0.02 to 0.52)	0.04
Fasting serum glucose, mmol/L	0.08 (−0.16 to 0.31)	0.52
Glycated hemoglobin (HbA1c), %	0.08 (−0.20 to 0.36)	0.59
Fasting insulin, pmol/L	−0.03 (−0.27 to 0.21)	0.80
eGFR (mL/min/1.73 m^2^)	0.26 (0.04 to 0.49)	0.02
Urea, mmol/L	−0.14 (−0.37 to 0.09)	0.23
Visceral adipose tissue index (VATI; g/m^2^)	−0.27 (−0.50 to −0.05)	0.017
Lean mass index (LMI) z-score	−0.34 (−0.58 to −0.09)	0.007
Total body less head (TLBH) bone mineral density (BMD) z-score	0.01 (−0.22 to 0.25)	0.91

**Table A2:** Associations between osteocalcin z-score and clinical, anthropometric, metabolic, and body composition parameters in boys.

Outcome Variable	β (95% CI)	*p* Value
Height z-score	0.23 (0.00 to 0.45)	0.046
Body weight z-score	0.05 (−0.18 to 0.28)	0.67
Body mass index (BMI) z-score	−0.07 (−0.29 to 0.16)	0.56
25-hydroxyvitamin D (25(OH)D_3_), ng/mL	0.15 (0.01 to 0.39)	0.04
Serum calcium, mmol/L	0.25 (0.03 to 0.47)	0.03
Parathyroid hormone (PTH), pmol/L	0.19 (−0.04 to 0.41)	0.10
Free triiodothyronine (fT3), pmol/L	0.24 (0.01 to 0.46)	0.04
Alanine aminotransferase (ALT), U/L	−0.16 (−0.38 to 0.07)	0.17
Leptin z-score	−0.04 (−0.27 to 0.19)	0.72
Total cholesterol, mmol/L	0.15 (−0.07 to 0.38)	0.18
High-density lipoprotein cholesterol (HDL-C), mmol/L	0.11 (−0.12 to 0.34)	0.33
Low-density lipoprotein cholesterol (LDL-C), mmol/L	0.16 (−0.07 to 0.39)	0.16
Triglycerides, mmol/L	−0.11 (−0.34 to 0.11)	0.34
Fasting serum glucose, mmol/L	−0.16 (−0.38 to 0.07)	0.18
Glycated hemoglobin (HbA1c), %	0.07 (−0.02 to 0.50)	0.07
Fasting insulin, pmol/L	−0.09 (−0.32 to 0.15)	0.47
eGFR (mL/min/1.73 m^2^)	0.32 (0.10 to 0.54)	0.005
Urea, mmol/L	0.09 (−0.14 to 0.32)	0.46
Visceral adipose tissue index (VATI; g/m^2^)	0.01 (−0.22 to 0.23)	0.96
Lean mass index (LMI) z-score	0.06 (−0.17 to −0.30)	0.60
Total body less head (TLBH) bone mineral density (BMD) z-score	0.22 (−0.0005 to 0.45)	0.0505

**Table A3:** Multivariable linear regression analysis of associations between osteocalcin z-score and selected biochemical outcomes in girls.

Outcome	β (95% CI)	*p* Value
25-hydroxyvitamin D (25(OH)D_3_), ng/mL	0.24 (0.03 to 0.44)	0.02
Serum calcium, mmol/L	0.06 (−0.16 to 0.28)	0.58
Free triiodothyronine (fT3), pmol/L	0.46 (0.24 to 0.68)	<0.001
Triglycerides, mmol/L	0.06 (−0.15 to 0.26)	0.58
eGFR (mL/min/1.73 m^2^)	−0.12 (−0.33 to 0.09)	0.47
Visceral adipose tissue index (VATI; g/m^2^)	−0.12 (−0.33 to 0.09)	0.27
Lean mass index (LMI) z-score	−0.08 (−0.31 to −0.15)	0.47

Adjusted R^2^ = 0.44, *p* < 0.001 for model. Multivariable linear regression models were fitted with OC z-score as the independent variable. Outcome variables included parameters that were statistically significant in univariable analyses. β coefficients represent the change in the outcome variable per 1 standard deviation increase in OC z-score. *p* values < 0.05 were considered statistically significant.

**Table A4:** Multivariable linear regression analysis of associations between osteocalcin z-score and selected biochemical outcomes in boys.

Outcome	β (95% CI)	*p* Value
Height z-score	0.21 (−0.02 to 0.44)	0.07
25-hydroxyvitamin D (25(OH)D_3_), ng/mL	0.21 (−0.005 to 0.43)	0.055
Serum calcium, mmol/L	0.09 (−0.14 to 0.31)	0.45
Free triiodothyronine (fT3), pmol/L	0.008 (−0.24 to 0.26)	0.95
eGFR (mL/min/1.73 m^2^)	0.23 (−0.02 to 0.48)	0.07

Adjusted R^2^ = 0.14, *p* < 0.001 for model. Multivariable linear regression models were fitted with OC z-score as the independent variable. Outcome variables included parameters that were statistically significant in univariable analyses. β coefficients represent the change in the outcome variable per 1 standard deviation increase in OC z-score. *p* values < 0.05 were considered statistically significant.
